# CRISPR Cas9 in Pancreatic Cancer Research

**DOI:** 10.3389/fcell.2019.00239

**Published:** 2019-10-18

**Authors:** Hai Yang, Peter Bailey, Christian Pilarsky

**Affiliations:** Department for Surgical Research, Universitätsklinikum Erlangen, Erlangen, Germany

**Keywords:** pancreatic cancer, CRISPR/Cas9, gene editing, knock out and knock in, library screening

## Abstract

Pancreatic cancer is now becoming a common cause of cancer death with no significant change in patient survival over the last 10 years. The main treatment options for pancreatic cancer patients are surgery, radiation therapy and chemotherapy, but there is now considerable effort to develop new and effective treatments. In recent years, CRISPR/Cas9 technology has emerged as a powerful gene editing tool with promise, not only as an important research methodology, but also as a new and effective method for targeted therapy. In this review, we summarize current advances in CRISPR/Cas9 technology and its application to pancreatic cancer research, and importantly as a means of selectively targeting key drivers of pancreatic cancer.

## Introduction

According to the data provided by the North American Association of Central Cancer Registries (NAACCR), pancreatic cancer is the 12th most common cancer with the 5th worst prognosis in the United States. It is estimated that there will be 56,770 new cases of pancreatic cancer and 45,750 pancreatic cancer-related deaths in the United States in 2019. Compared to other common cancers, pancreatic cancer has not seen a significant improvement in patient outcomes, with a 9% 5-year relative survival (Siegel et al., 2019). Surgery, radiation therapy and chemotherapy are still the main treatment options for pancreatic cancer, but there is now considerable effort in identifying better treatment strategies for pancreatic cancer, such as targeted therapy, immune therapy and potentially CRISPR/Cas9 directed gene therapy.

Currently CRISPR/Cas9 (clustered regularly interspaced short palindromic repeats and CRISPR-associated protein 9) is emerging as a powerful gene-editing tool with potential in precision medicine. CRISPR repetitive sequences were first observed by Ishino et al. (1987), with subsequent work performed by Jinek et al. (2012) proving that an endonuclease can be directed to cleave target DNA by a two-RNA structure. Since then, CRISPR/Cas9 technology has rapidly evolved, driving incredible progress in research and clinical applications. Compared to the other gene-editing technologies, such as meganucleases (MNs), zinc finger nucleases (ZFNs) and transcription activator-like effector nucleases (TALENs), CRISPR/Cas9 technology has lower cost, higher efficiency and is less complex in its application (Osborn et al., 2016).

## Background of CRISPR/Cas9

CRISPR (clustered regularly interspaced short palindromic repeats) were first described in 1987 by Ishino et al. (1987) who identified highly conserved nucleotide sequences exhibiting a 14 bp dyad symmetry at the 3′-end flanking region of the isozyme-converting alkaline phosphatase (iap) gene in *Escherichia coli.* However, the function and biological importance of CRISPR sequences were not fully understood until 2007, when Barrangou et al. (2007) exposed Streptococcus thermophilus to phage and sequenced the resultant phage-resistant variants. Analysis of the variant DNA revealed that the bacteria had gained new CRISPR spacers that were derived from the phage genome. The identification of CRISPR sequences in bacterial genomes subsequently led to the identification of a set of homologous genes referred to as CRISPR and associated (*cas*) genes that together comprise the CRISPR locus. The insertion of CRISPR-Cas in the genomes of bacteria infected with virus suggested that CRISPR-Cas may provide resistance against phages and that resistance to infection might be enhanced or decreased by inserting or deleting spacer-phage sequence.

Jinek et al. (2012) in 2012 were the first to demonstrate that the CRISPR-Cas system could produce a mature CRISPR RNA (crRNA) and base-paired *trans-*activating crRNA (tracrRNA) that together form a two-RNA hybrid structure. This two-RNA structure could guide CRISPR-associated protein Cas9 to specific DNA sequences and generate double-stranded (ds) breaks in target DNA. In 2013, Cong et al. (2013) and Mali et al. (2013) successfully edited the DNA sequences of the EMX1, PVALB, PPP1R12C genes in human and the Th gene in mouse using the CRISPR/Cas9 system. This was the first demonstration of the power of the CRISPR-Cas9 system to edit the genomes of eukaryotic cells. Since then, a wealth of research has improved the CRISPR/Cas9 gene editing tool’s specificity, orthogonality and multiplexibility in various species, and has led to the development of new omics applications.

CRISPR/Cas9 technology has been rapidly promoted and applied in the: generation of animal models; gene function research; multiplexed mutations; and chromosome rearrangements. Compared to traditional gene editing tools such as MNs, ZFNs, TALENs and so on, CRISPR/Cas9 technology provides the advantage of lower cost, higher efficient and greater simplicity (Osborn et al., 2016).

## Application of CRISPR/Cas9 in Pancreatic Cancer

### CRISPR/Cas9 Gene Editing in Pancreatic Cancer

An homologous guide RNA can guide the cas9 nuclease to target a specific site in the genome. When cas9 is guided to the target DNA sequence and there is a Protospacer Adjacent Motif (PAM) sequence downstream, it will cut both strands of the genome at that location. The cells then repair the break using two distinct mechanisms: (1) imprecisely by using the non-homologous end joining (NHEJ) repair pathway, which causes the insertion or deletion of bases; or (2) accurately by the homology-directed repair (HDR) pathway. Based on existing homologous DNA sequences, the HDR pathway can repair DNA damage accurately by mediating a strand-exchange process involving the provided DNA sequence as a repair template to insert a matching DNA sequence into the break. By inducing NHEJ or HDR, CRISPR/Cas9 knockouts or knockins can be achieved by deleting, replacing or adding genetic sequences.

CRISPR/Cas9 system is widely used in pancreatic cancer research to knock-out genes implicated in disease progression. Watanabe et al. (2018) used CRISPR/Cas9 to knock out KDM6A in human Pancreatic ductal adenocarcinoma (PDAC) cell lines to demonstrate that KDM6A-deficient cells exhibit an aggressive phenotype. Pessolano et al. (2018) and Belvedere et al. (2016) showed that, CRISPR/Cas9-directed knock-out of ANXA1 in Mia PaCa2 cells, resulted in the secretion of fewer extracellular vesicles and a weak motile phenotype. After knock out of GALNT3 in Capan1 cells, Barkeer et al. (2018) found that cells formed fewer tumorspheres, lost their ability to self-renewal, and migrate. Yuza et al. (2018) demonstrated that knock out of SphK1in PAN02 cells resulted in increased proliferation and migration. C1GALT1 was knocked out of PDAC cells by Chugh et al. (2018) with CRISPR/Cas9 technology, and the cells showed increased growth, migration, tumorigenicity, metastasis and expression of Tn and sTn. There are also some other researches in pancreatic cancer based on the same strategy by knocking out specific gene of pancreatic cancer cells, then observe the changes in different phenotypes (Table 1; Belvedere et al., 2016; Hesler et al., 2016; Muniyan et al., 2016; Rao et al., 2016; Vorvis et al., 2016; Ayars et al., 2017; Harada et al., 2017; He et al., 2017; Lal et al., 2017; Muzumdar et al., 2017; Yu et al., 2017; Zhang et al., 2017; Barkeer et al., 2018; Chugh et al., 2018; Liu et al., 2018a, b, 2019; Pessolano et al., 2018; Santoro et al., 2018; Wang et al., 2018; Watanabe et al., 2018; Yasunaga et al., 2018; Yuza et al., 2018; Abdalla et al., 2019; Hwang et al., 2019; Li et al., 2019b, c; Stock et al., 2019). Collectively, these studies show that CRISPR/Cas9 is a very powerful gene editing tool for exploring the function of defined genes in cell signaling pathways, proliferation, migration, invasion and potentially chemotherapy resistance of pancreatic cancer. By using CRISPR/Cas9 technology to knock out target genes and characterize phenotypic changes, we can develop a better understanding of biological roles played by target genes, identify potential treatment targets and/or better treatment strategy for pancreatic cancer. But there is still a long way to go before CRISPR/Cas9 technology can be used as an effective treatment strategy in the clinic.

**TABLE 1:** Recent researches of gene function in pancreatic cancer by CRISPR/Cas9 knockouts of pancreatic cancer cells.

**sgRNA target**	**Cell lines**	**Effect**	**References**
KDM6A	BxPC3, PANC1	proliferation↑, migration↑, invasion↑	Watanabe et al., 2018
ANXA1	MIA PaCa-2	migration↓, invasion↓, extracellular vesicles↓	Pessolano et al., 2018; Belvedere et al., 2016
SphK1 SphK2	PAN02	proliferation↑, migration↑, survival of mice↓ proliferation↓, migration↓, survival of mice↑	Yuza et al., 2018
GALNT3	Capan1	proliferation↓, migration↓	Barkeer et al., 2018
C1galt1	KPC, KPCC	proliferation↑, migration↑, tumorigenicity↑, metastasis↑	Chugh et al., 2018
ARID1A	HPDE	H2K27ac marks↑	Wang et al., 2018
GPRC5a	MIA PaCa-2, TB32047	proliferation↓, migration↓, chemotherapy resistance↓	Liu et al., 2018a
ECT2	MIA PaCa-2	proliferation↓, migration↓	Liu et al., 2018b
ATG12	MIA PaCa-2, AR42J	expression of duct cell markers↑, expression of acinar cell markers↓	Yasunaga et al., 2018
KRAS	A13,8988T, PANC1, KP-4, MM1402, PACO9, PACO19	exhibit PI3K dependence	Muzumdar et al., 2017
HuR	MIA PaCa-2	apoptosis↑, unable to engraft tumors *in vivo*	Lal et al., 2017
	Hs 776T	unable to engraft tumors *in vivo*	
	HCT116	proliferation↓	
Rab11-FIP4	PANC-1	proliferation↓, invasion↓, metastasis↓	He et al., 2017
IRAK4	PANC-1, Pa01c	anchorage-independent (AI) growth↓	Zhang et al., 2017
CYR61	MIA PaCa-2, PANC1	hENT1 and hCNT3 expression↑, cellular uptake of gemcitabine↑, gemcitabine-induced apoptosis↑	Hesler et al., 2016
MUC16	Capan1	expression of Thomsen-Friedenreich (TF/T) and Thomsen-nouvelle (Tn) carbohydrate antigens↓	Muniyan et al., 2016
FOXA2	PANC-1	tumorigenicity↑, aggressiveness↑	Vorvis et al., 2016
GCNT3	MIA PaCa, BxPC-3, PANC-1	proliferation↓, spheroid formation↓	Rao et al., 2016
CTTN	PANC-1, BxPC-3	migration↓, invasion↓	Stock et al., 2019
CRABP-II	PANC-1	migration↓, invasion↓, expression of interleukin 8 (IL-8), MMP-2 and MMP-14↓	Yu et al., 2017
HO-1	Capan-1	Under hypoxia, proliferation↓, sensitivity to gemcitabine↑	Abdalla et al., 2019
WNT5B	PANC-1	migration↓, invasion↓	Harada et al., 2017
CDH17	Panc02-H7	Proliferation, colony formation and motility↓	Liu et al., 2019
EI24	MIA PaCa-2	Proliferation↓	Hwang et al., 2019
HO1	CD18/HPAF, COLO 357, Capan-1, Mia PaCa-2	under hypoxia, proliferation↓, sensitive to gemcitabine↑	Abdalla et al., 2019
IL2RG	TB32043, bkpc58	Tumor growth in mice↓, JAK3 expression in orthotopic tumors↓	Ayars et al., 2017
HIF-1α	BxPC-3	Metastasis↓	Li et al., 2019b
MEKK3	Panc-1, AsPC1	EMT and cell migration↓, tumor growth↓, mice overall survival↑	Santoro et al., 2018
ERBB2	HPAFII, CAPAN1, Mia PaCa-2 (all ERBB2-amplified)	ERBB2 signaling, colony formation, anchorage-independent growth and tumor xenograft formation↓	Li et al., 2019c

*↑, up-regulation; and ↓, down-regulation.*

Gene knockins by CRISPR/Cas9 are also quite important for exploring gene functions in tumorigenesis and development and commonly used for building specific gene expression stem cells and animal models in recently years (Yang et al., 2013; Platt et al., 2014; Ma et al., 2017; Li et al., 2019a). But due to the complicated procedure and time consumption, it is still more common to generate gene expressing cells by building gene expression vectors and transducing the vector into cells via retrovirus or lentivirus in pancreatic cancer research (Li et al., 2019c; Sharma et al., 2019; Watanabe et al., 2019).

### CRISPR Pooled Library Screening in Pancreatic Cancer

Using a sgRNA library to perform loss-of-function (LoF) phenotypic screens is a powerful way to identify novel protein functions in specific growth conditions. The main strategy of CRISPR pooled library screening involves the use of thousands of plasmids in one CRISPR pooled library, with each plasmid containing an sgRNA targeting a specific gene. The sgRNAs first needs to be delivered into a sufficient number of cells via lentivirus or retrovirus transduction. The transduced cells are then grown under specific conditions to select a phenotype of interest. After the selection, the genomic DNA is isolated from the cells, and next-generation sequencing (NGS) performed. The NGS data are then mapped to a pre-complied library comprising the gene-specific gRNAs and the results analyzed by computational tool such as MAGeCK (Model-based Analysis of Genome-wide CRISPR-Cas9 Knockout) (Li et al., 2014). Genes responsible for the observed phenotype are then identified by comparing control and selected gRNAs.

Several studies have now used CRISPR pooled library screening in pancreatic cancer cells (Table 2; Steinhart et al., 2017; Wang et al., 2017; Szlachta et al., 2018; Bakke et al., 2019; Sarr et al., 2019). Bakke et al. (2019) transduced PANC-1-cas9 cells with human sgRNA library Brunello and selected the cells with 100 nM gemcitabine for 6 days. PSMA6 was identified as a top hit after comparing NGS results obtained for gemcitabine selected samples and control samples. PSMA6 inhibition was subsequently shown to result in apoptosis and reduced spheroid formation. In Steinhart et al. (2017), a TKO gRNA library was transduced into HPAF-II-Cas9 cells, and cells screened at different time points to identify the set of fitness genes required for proliferation. They used the BAGEL algorithm (Hart and Moffat, 2016) to calculate a log Bayer factor (BF) for each gene. This analysis identified several core components of the Wnt pathway, including WLS, CTNNB1, TCF7L2, LRP5, and PORCN, and 3 Frizzled (FZD) receptors and Wnts encoding genes, FZD5, WNT7B, WNT10A, as essential genes for HPAF-II cell proliferation. These results were subsequently validated in AsPC-1 and PaTU8988S cell lines, identifying the Wnt pathway as an important mediator of pancreatic cancer cell proliferation. These screen results also revealed that the proliferation and/or survival of RNF43-mutant PDAC cells are selectively dependent on Wnt-β-catenin signaling. Wang et al. (2017) also performed genome scale CRISPR-Cas9 knockout screens in PATU8902 and PATU8988T cell lines to identify genes capable of promoting proliferation or survival in the context of MAPKi by deletion. They transduced the GeCKOv2 library into PATU8902 cells, and Avana library into PATU8988T cells. PATU8902 cells were treated with MEK1/2 inhibitor trametinib 100 nM and PATU8988T cells were treated with 10 nM trametinib. Both cell lines were treated with trametinib for 14 days. Genomic DNA was then isolated and NGS performed. CIC and ATXN1L were identified by comparative analysis, indicating that loss of both CIC and ATXN1L can reduce sensitivity to trametinib treatment *in vivo*.

**TABLE 2:** Recent researches of CRISPR pooled library screening in pancreatic cancer.

**Hit genes**	**Effect**	**Cell line**	**Library**	**Gene/sgRNA number**	**Selection condition**	**References**
PSMA6	apoptosis↑, spheroid formation↓	PANC-1	Human sgRNA library Brunello (addgene No. 73178)	19114/76441	100 nM gemcitabine for 6 days	Bakke et al., 2019
Wnt pathway genes, FZD5	Proliferation↓	HPAF-II, AsPC-1, PaTu8988S	Toronto KnockOut (TKO) CRISPR Library (addgene No. 1000000069)	17232/91320	different time point (Day 15, 27, 31, 35)	Steinhart et al., 2017
CIC, ATXN1L	sensitivity to trametinib↓	PATU8902	GeCKOv2 library (addgene No. 1000000049)	19050/123411	100 nM trametinib for 14 days	Wang et al., 2017
		PATU8988T	Avana library Doench et al., 2016	18675/110257	10 nM trametinib for 14 days	
CENPE, RRM1	sensitivity to trametinib↑	PDX366	Nuclear proteins gRNA sub-pool library (addgene No. 51047)	7114/73151	MEK and CENPE inhibitor treatment in 6–8-week-old mail athymic nude mice	Szlachta et al., 2018
DCK, DCTPP1	sensitivity to NUC-1031, gemcitabine↓	Mia PaCa-2, A2780	GeCKOv2 library (addgene No. 1000000049)	19050/123411	15 nM gemcitabine or 65 nM NUC-1031 for 14 days or 21 days	Sarr et al., 2019

*↑, up-regulation; and ↓, down-regulation.*

These studies successfully demonstrate that novel gene functions can be identified using CRISPR pooled library screening with specific selection conditions, thereby providing a very powerful methodology for identifying therapeutic vulnerabilities in pancreatic cancer cells and pointing the way toward new treatment strategies. But there are also several factors that can affect the accuracy of the screening results. Based on different size of libraries, different number of cells are required to be transduced for maintaining the coverage of the libraries. Weak selection conditions may provide more comprehensive results, but will also produce false positive which may not be causative in phenotypic changes. Different selection methods may also drive experimental variability. The deep sequencing quality and the selection of data analysis tools will also play very important roles in screening accuracy. Therefore, results still need to be validated using orthogonal methods.

### Current Status of CRISPR/Cas9 Gene Therapy for Pancreatic Cancer

In pancreatic cancer gene therapy, there are several current developments including gene-based tumor cell sensitization to chemotherapy, vaccination, and adoptive immunotherapy (Rouanet et al., 2017). But recently the CRISPR/Cas9 genome editing method has been utilized in several pancreatic cancer studies due to its safety and efficacy. CRISPR/Cas9 gene therapy could be potentially used both *ex vivo* and *in vivo*. In *ex vivo* therapy, cells could be isolated and modified outside of the body, and then transplanted back into the body. In *in vivo* therapy, genetic materials could be directly injected into the body (Song, 2017). Chiou et al. (2015) built *in vivo* pancreatic cancer models via retrograde pancreatic ductal injection of either adenoviral-Cre and lentiviral-Cre vectors. This technique could establish new therapies for pancreatic cancer. Accordingly, CRISPR/Cas9 technology is providing great hope for the treatment of monogenic diseases, degenerative diseases and HIV infection, and several CRISPR/Cas9 clinical trials have been performed, but there is still a long way to go before the CRISPR/Cas9 technique is used to treat pancreatic cancer patients. Several challenges still need to be considered. For example, which crucial gene(s) should be targeted? And the accuracy, efficiency and safety need to be increased to meet the requirement of clinical application.

## Limitations of the Crispr/Cas9 Application

CRISPR/Cas9 is widely applied not only in pancreatic cancer research, but also other cancer settings to further our understanding of disease progression, identify mechanisms of drug resistance and to uncover potential therapeutic vulnerabilities. However, there are still some limitations to this technology that need to be considered before CRISPR/Cas9 is used in the clinical practice. Potential off-target effect are a major concern and need to be minimized to ensure safety. Considerable effort has been made to reduce off-target effects. Ran et al. (2013) combined the D10A mutant nickase Cas9 (Cas9n) with a pair of offset sgRNAs which are complementary to opposite strands of the target site. This approach improved the specificity of CRISPR/Cas9 editing without sacrificing on-target cleavage efficiency. Shen et al. (2014) used Cas9(D10A) or Cas9(H840A) to perform AR-A and AR-B gene editing in mouse fibroblast cells. T7EN cleavage assay and TA cloning were performed to detect the modification of the AR gene, and off-target effects were not found. Tsai et al. (2014) demonstrated that dimeric RNA-guided *Fok*I Nucleases (RFNs) could recognize extended sequences and edit endogenous genes with high efficiencies.

## Conclusion and Outlook

Compare to the other gene-editing technologies, CRISPR/Cas9 technology involves lower costs, higher efficiency and simplicity of use. It is widely used to characterize gene function(s) under specific selection conditions and is gaining traction in pancreatic cancer research. But there is still a long way before this technology can be used as a selective gene therapy in the clinics. Improving the specificity of cas9 gene editing, reducing off-target effects and increasing the efficiency of cell transduction and cell targeting are all important challenges that need to be overcome. As a maturing gene editing technology, CRISPR/Cas9 represents a “game changer” for scientific research and an exciting targeted therapy for pancreatic cancer.

## Author Contributions

HY wrote the manuscript and researched the publication landscape. PB revised the manuscript. CP devised the idea.

## Conflict of Interest

The authors declare that the research was conducted in the absence of any commercial or financial relationships that could be construed as a potential conflict of interest.
